# A Data Compression Algorithm for Wireless Sensor Networks Based on an Optimal Order Estimation Model and Distributed Coding

**DOI:** 10.3390/s101009065

**Published:** 2010-10-11

**Authors:** Peng Jiang, Sheng-Qiang Li

**Affiliations:** Institute of Information and Control, School of Automation, Hangzhou Dianzi University, Hangzhou 310018, China; E-Mail: lisqou@163.com (L.S.-Q.)

**Keywords:** optimal order estimation, distributed coding, data compression

## Abstract

In many wireless sensor network applications, the possibility of exceptions occurring is relatively small, so in a normal situation, data obtained at sequential time points by the same node are time correlated, while, spatial correlation may exist in data obtained at the same time by adjacent nodes. A great deal of node energy will be wasted if data which include time and space correlation is transmitted. Therefore, this paper proposes a data compression algorithm for wireless sensor networks based on optimal order estimation and distributed coding. Sinks can obtain correlation parameters based on optimal order estimation by exploring time and space redundancy included in data which is obtained by sensors. Then the sink restores all data based on time and space correlation parameters and only a little necessary data needs to be transmitted by nodes. Because of the decrease of redundancy, the average energy cost per node will be reduced and the life of the wireless sensor network will obviously be extended as a result.

## 1. Introduction

Wireless sensor networks can be broadly applied in various areas such as environmental monitoring, medical care, intelligent homes, transportation, military fields, *etc*. Data compression algorithms for wireless sensor networks aim to find an efficient way to compress data for reducing node energy costs and improving the synthesized ability of the whole system. Meanwhile, the accuracy must be guaranteed when data is being decoded.

Many new algorithms have been proposed recently, such as the self-based regression algorithm proposed by Deligiannakis, which first splits the recorded series into intervals of variable length [1]. Then these intervals will be encoded based on an artificially constructed base signal, so the base signal and the extent of the piece-wise linear recorded series are critical factors of the algorithm. A multidimensional sequential pattern mining over data streams algorithm as proposed by Rassi and Plantevit in [2] scans the temporal series record and realizes data coding based on pattern frequency. Meanwhile, multidimensional sequential patterns extracted from data can be used to compress data. Because the data need to be scanned, the memory and energy cost of nodes must be larger and the real time ability of the network will be affected. A data compression algorithm based on route choosing proposed by Pattem and Krishnamachari combines routing and data compression [3]. The algorithm reduces data space redundancy by balancing the data relativity of adjacent nodes and the size of clusters. Then the average energy cost of node will be decreased and the longevity of system will be extended. However, the algorithm needs to set accurate positions for nodes and the distance between nodes should be same, which is hard to realize in practice. Meanwhile, the relativity of data decides the performance of the algorithm and getting relativity parameters is hard in actual applications. The Group-Independent Spanning Tree algorithm proposed by Lujun Jia and Guevara also aims to reduce the space redundancy of data [4], by contructing a complete data transmission structure, but their discussion of algorithm parameters and eliminating redundancy within clusters is insufficient.

Zhou and Lin have proposed a distributed spatial-temporal data compression algorithm based on a ring topology wavelet transformation [5], which supports a broad scope of wavelet transformations and is able to efficiently decrease spatial-temporal redundancy, but the algorithm may be too complex for nodes because of the wavelet transformations. Lin and Vana proposed an online information compression algorithm [6], which first divides data obtained by nodes into different lengths of shorter data, then a dictionary can be composed according to the shorter data and updated with the increased data obtained by nodes. The algorithm can reduce the average energy cost per node and improve the accuracy of the restored data, however, the course of updating the dictionary makes the algorithm more complex than the self-based regression algorithm discussed above. The distributed structure tree depression algorithm (abbr. DSTD) proposed by Chou and Petrovic in [7] explores the spatial-temporal relativity that exists in data and computes the relativity parameters in the sink. Then the sink restores data according to relativity parameters and part of the original data transmitted from the nodes, so the nodes’ energy cost can be reduced to some extent. However, the number of data groups transmitted from nodes to sink is not defined when estimating relativity parameters. If a node transmits too much data for estimating relativity, some redundant parameters will be imported, then the node energy will be wasted and accuracy will be affected. On the other hand, if data transmitted for estimating relativity is not enough, the accuracy of data restored in the sink will be seriously affected.

This paper proposes a cluster optimal order estimation distributed structure tree depression algorithm (abbr. COOE-DSTD). The main idea of the algorithm is injecting the theory of optimal order estimation model into the field of data compression for wireless sensor networks, which has not been tried by others before. The simulation results demonstrate that the combination of optimal order estimation and distributed coding is effective for improving the data compression algorithm of wireless sensor networks. According to optimal order estimation we define the number of groups of data transmitted by nodes for obtaining relativity parameters, so redundant parameters can be prevented. At the same time, the wireless sensor network is divided into clusters, which not only improves the efficiency of the sink to deal with data, but also strengthens the ability of the sink to locate the position of exceptions, so the monitoring ability of the whole system will be greatly improved. The second part will introduce distributed coding theory and the optimal order estimation model imported by COOE-DSTD. The basic structure and flow chart of the DSTD algorithm and the COOE-DSTD algorithm will be given in the third section. The fourth part demonstrates the performance of the COOE-DSTD algorithm through comparison with DSTD from the point of view of average energy cost per node, signal to noise ratio and the ratio of the above two factors. Meanwhile, relative simulations are shown and analyzed. Conclusions of the paper and prospects for future work are offered in the last section.

## 2. Distributed Coding and Optimal Order Estimation

Data obtained by nodes in wireless sensor networks includes spatial-temporal relativity, which is the basis to introduce distributed coding in the DSTD and COOE-DSTD algorithms. Meanwhile, optimal order estimation is introduced in COOE-DSTD. Now a necessary explanation of distributed coding and optimal order estimation is given.

**Figure 1.**
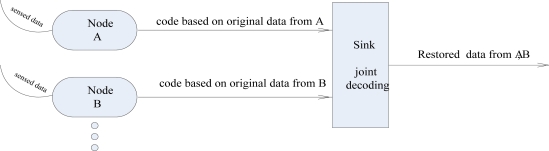
Distributed coding.

Distributed coding is one kind of asymmetric coding. First let us take the situation of two nodes as an example, as Figure 1 shows. Node A transmits original data or code which is coded according to data already obtained by A. So does node B. In the sink, if original data from A is obtained, the current data from A can be restored by code from A.

In the same way, current data from B can be restored according to code from B and data already restored from A. If data obtained by nodes are discrete independent identically distributed sequence, both nodes A and B can restore their original data as better as they don’t know the relativity parameters between A and B. When extending two nodes to N nodes, the sink obtains partial original data and code from nodes and computes relativity parameters. Then combined with these relativity parameters, the sink can restore all original data. Distributed coding derives from the sympatric data source coding theory which was proposed by Slepian and Wolf [8].This theory demonstrates that if correlated random variables X, Y obey arbitrary possibility distribution *p(x,y)*, no matter whether the relativity between X and Y is known by the coder or not, the compression performance will be equal. When the relativity between X and Y is unknown, X can be coded with *H(x|y)* bits. And *H(x|y)* can be demonstrated as:

(1) $$H(x|y)=-\sum_{y}P_{Y}(y)\sum_{x}P_{X}(x|y)\log P_{X}(x|y)$$

In Equation (1), *H(x|y)* stands for the uncertainty of variable X when Y is given. Now let us take an example to explain the theory in detail. Suppose variables X, Y are distributed with equal possibility in a data set whose elements consist of three bits. Variables X, Y obey *d_H_(X, Y)* ≤ 1. That is to say, X, Y with equal possibility distribute in set {000, 101, 011, 110} and {001, 010, 100, 111} respectively. The term *d_H_(m,n)* stands for the Hamming distance function. When Y is known by both coder and decoder, X can be coded by two bits, which stands for the uncertain information of X. However, supposing only the decoder knows Y, X still can be coded by two bits. This can be achieved by dividing the set into four cosets. They are coset1 = (000,111); coset2 = (001,110); coset3 = (010,101); coset4 = (011,100). Only two bits are needed to code four cosets. If the coset including X is known together with Y, and X will be decoded lossless. According to the above illustration, some nodes need to transmit three bits while other nodes only need to transmit two bits, so the coding mode is asymmetric and the energy cost of nodes may be different. We introduce the time period rotation method to balance the energy cost of all nodes when applying coding theory to decrease data redundancy. Besides, the way of coding described above can’t support variable compression ratios and sinks will construct a special structure tree to solve the problem, which will be illustrated in detail in the following section.

The course of getting data through nodes in wireless sensor networks is considered a stationary stochastic process in this paper. The optimal order estimation can be explained as follows: as known to all, the performance of a given autoregressive model is up to the practical process, the number of samples, the estimation algorithm and the order selection criterion. The finite sample criterion has given an empirical estimation based on the residual energy statistical average and an autoregressive estimation algorithm of predicted variance, which makes the performance of the finite sample criterion dependent on the adopted estimation method. As two important criteria, both special the finite sample information criterion (abbr. FSIC) and the combined information criterion (abbr. CIC) have considered the increasing residual energy with the increase of model order [9], which is ignored by other criteria, so compared with FSIC and CIC, other criteria often get over fit order because they only take the expectation of the logarithm of the residual energy as the function of the order model.

Now we first illustrate FSIC and CIC in detail. For a practical stochastic process *AR(R)* [10]:

(2) $$y_{n}+a_{1}y_{n-1}+\cdots a_{R}y_{n-R}={ɛ}_{n}$$

We set the model of *AR(R)* as *AR(g)* as follows:

(3) $$y_{n}+{\hat{a}}_{1}y_{n-1}+\cdots {\hat{a}}_{g}y_{n-g}={\hat{ɛ}}_{n}$$

In Equation (3) *ε_n_* stands for stationary stochastic process whose mean and variance are 0 and $\delta_{ɛ}^{2}$, respectively. The last parameter *â_g_* is called the reflection coefficient. The variance coefficient *V* (*d*, •) is the basic part of the finite sample criterion for the AR model. The term *d* stands for model order while “•” stands for estimation method in *V* (*d*, •). This paper has adopted the estimation method of Burg, which is a correlation estimation method. *V* (*d*, •) is an approximate estimation of the empirical variance of the reflection coefficient when order *d > R*. Meanwhile, *V* (*d*, •) is also an approximate estimation of the empirical variance of parameter *â_g_* when fitting model *AR (g)* by the least squares method. If the estimation model order is larger than the practical stochastic process, the variance of the reflection coefficient will be set as 1/*N*, where *N* stands for sample capacity. If we adopt the above estimation method for the *AR (g)* model, we will get a *d* order empirical variance estimation equation as follows [10]:

(4) V(d,Burg)=1/(N+1−d)

If the *d* order empirical expectation of *AR(d)* model is negative or zero, we will get *V*(0,•) = 1/*N*. The above estimation will be accurate for practical stochastic process under the condition that *d > R*.

As the variance of ɛ̂_*n*_, $\delta_{\hat{ɛ}}^{2}$ is called residual variance *RES(g)* when data *y_n_* in Equation (3) is used to estimate parameter *â_d_*. $\delta_{\hat{ɛ}}^{2}$ is defined as the predicted error *PE(g)* when parameters *â_d_* and *y_n_* are mutually independent. If model order g is larger than or equal to the practical random order *R* and the Burg estimation method is adopted to estimate *AR(g)*, we will get approximate expressions of the residual variance *RES(g)* and predicted variance *PE(g)* as follows [10]:

(5) $$E\left\{\mathrm{RES}(g)\right\}\approx \delta_{ɛ}^{2}\prod_{d=0}^{g}\left\{1-V(d,•)\right\}\;g\geq R$$

(6) $$E\left\{\mathrm{PE}(g)\right\}\approx \delta_{ɛ}^{2}\prod_{d=0}^{g}\left\{1+V(d,•)\right\}\;g\geq R$$

Replacing *V* (*d*, •) in (5), (6) with the definite value of *V* (*d*, •) in (4) we can get an approximate expectation of *RES (g)* and *PE (g)*. So, both (5) and (6) can be fitted by the least squares method and relative proof can be found in [9]. The expectation of the ratio of *PE (g)* and *RES (g)* is introduced in the finite sample information criterion. We can get Equation (7) for order estimation [10]:

(7) $$\mathrm{FSIC}(g)=ln\{\mathrm{RES}(g)\}+\prod_{d=0}^{g}\frac{1+V(d,•)}{1-V(d,•)}-1$$

Compared with other criteria which just modify *ln {RES (g)}*, FSIC can reflect the main fluctuation of g/*N* under the condition of g/*N* > 0.1. That is to say, FSIC can obtain an optimal order better than other criteria under the situation of g/*N* > 0.1. However, if the optimal model order is small, it will be hard for FISC to give a good estimation. CIC can solve the above problem very well. Because the punishment factors and merits of FISC are adopted by CIC at the same time. Punishment factor is suitable for a low order optimal estimation while FISC is appropriate for a low order optimal estimation. No matter whether the optimal order of the model is high or low, CIC can obtain suitable order estimation. The definition of CIC is as follows [10]:

(8) $$\mathrm{CIC}(g)=\ln\{\mathrm{RES}(g)\}+\prod_{d=0}^{g}\frac{1+V(d,•)}{1-V(d,•)}-\min\{1,3\sum_{d=0}^{g}V(d,•)\}$$

If the optimal order of model is low, the CIC can avoid optimal order being estimated too low through a punishment factor $3\sum_{d=0}^{g}V(d,•)$. The CIC can switch to the form of FSIC automatically to avoid the optimal order being estimated too high when the optimal order of model is high, so the combined information criterion is used to estimate the optimal order in the following algorithm proposed in this paper.

## 3. Descriptions and Flow Chart of the Algorithm

A simple description of the basic theory referred in the proposed algorithm is given above. Now we will illustrate the algorithm in detail. Based on distributed coding theory, DSTD first let nodes transmit N/3 groups’ original data to the sink, then the sink constructs the structure tree and computes prediction relativity parameters and number *i* based on original data. N stands for the number of data groups sensed by nodes in a complete cycle of the algorithm and *i* stands for the number of bits which is needed for nodes to compress the original data. By contrast, in the COOE-DSTD algorithm proposed in this paper all nodes transmit the obtained data to the sink and then the sink will judge whether it is getting the optimal order or not through the combined information criteria once the one time data transmission is finished by every node. If the order is not optimal and the number of transmission times is smaller than N/3, nodes will continue to transmit original data, or the sink will compute the initial predictive coefficients and construct the structure tree. The distributed coding method is also used in the COOE-DSTD algorithm, so the sink needs to appoint a node to transmit its original data. The node which transmits the original data expends more energy than those transmitting compressed code, therefore the sink will allocate a node in turn to transmit its original data to balance the energy consumption of all nodes. When a node receives the compression instruction *i*, a modulo operation of the original data (analog to digital conversed) by 2*^i^* will be made in the node, then the original data is compressed into *i* bits code, which will be received by the sink. The code will map a little data sequence in the structure tree built in the sink. With original data sensed by the allocated node and the predicted parameters computed above, we can restore different original data mapped by different *i* bits code. If all nodes’ original data of the same cycle are restored, combining with new original data, the sink will update the predicted parameters and compute the value of *i* for next cycle. The cycle continues and new data will be restored cycle by cycle.

**Figure 2.**
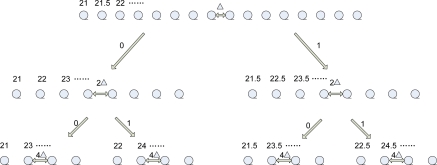
Structure tree.

Both the DSTD algorithm and COOE-DSTD algorithm adopt the same theory to get predicted correlation coefficients and the same way to establish the structure tree for getting the coding instruction *i*. Now it is necessary to give some explanation about the structure tree, predicted correlation coefficients and the coding instruction *i* referred above. In the DSTD algorithm we start with the average value of N/3 groups of original data for building the structure tree and extend the average original data from both sides by the interrupt of *Δ*, which can affect the accuracy of the algorithm. The range of extension is up to the practical application. Correspondingly, in the COOE-DSTD algorithm the establishment of the structure tree begins from the average value of the first group of original data. If we split the sequence into odd and even, then we will get two sub-sequences, which are interrupted by 2*Δ*. The sub-sequences can be split in the same way. After *i* times splitting, we can get some sub-sequences. Every sub-sequence maps to a code, which includes *i* bits. Let us use an example to explain the above process. Suppose all nodes monitor temperature and the average of the first N/3 groups data is 25 cent degrees. We set *Δ* = 0.5 and the range of extension is from 21 cent degrees to 28.5 cent degrees. If the range is exceeded, we will get an exception signal, which will be transmitted to the sink immediately. According to the above description, we can construct a structure tree as follows:

The *i* bits code mapped to the last row sub-sequence in Figure 2 is 00,01,10,11. If the sink gets an *i* bits code, it will map a sub-sequence. Combining with an estimation value based on prediction parameters, the sink can ascertain a value nearest to the corresponding original data in the sub-sequence. Then we can estimate original data.

Because of the existence of spatial-temporal relativity in the original data, we can construct a predictive model in the sink. That is to say, we can estimate the original data for the next moment based on the obtained data. It is necessary to declare that the node clustering operation is used after getting the optimal order of the estimation model in the COOE-DSTD algorithm. Both spatial relativity and temporal relativity of the original data obtained from nodes are relative to the optimal order, so the sink can divide all nodes into some clusters based on the value of the optimal order in the COOE-DSTD algorithm. For the first step, every node broadcasts its residual energy to nearby nodes. Secondly, every node compares its own residual energy with the received energy messages from nearby nodes. Thirdly, if one node detects that its energy is larger than that of a certain number of nodes (the number is determined by the value of optimal order), it will broadcast to those nearby nodes (those nodes’ remain energy are lower than that of the broadcaster) that it is their cluster head node. Many border nodes may be found after clustering, and these nodes can join a nearby cluster randomly. In this way, those nodes that have spatial relativity can be clustered together naturally. Then the Compression instruction will be computed by the sink for every node within a cluster. By contrast, in the DSTD algorithm the sink will compute predicted correlation coefficients and compression instructions within the whole sensor network, which will be more complex and more time and energy will be spent.

Supposing node j obtains some original data $X_{T}^{j}$ at moment T. We can get $Y_{T}^{j}$ through linearly fitting $X_{T}^{j}$ as follows [7]:

(9) $$Y_{T}^{j}=\sum_{k=1}^{W}\alpha_{k}X_{T-k}^{j}+\sum_{t=1}^{S}\beta_{t}X_{T}^{t}$$

In Equation (9), $\sum_{k=1}^{W}X_{T-k}^{j}$ stands for W continuous original data obtained by node *j* before moment T and $\sum_{t=1}^{S}X_{T}^{t}$ stands for S original data obtained by different nodes nearby node *j* at the moment T. W, S stand for the number of values obtained by the same node in different moments and different nodes in the same moment, respectively. So how to obtain proper predictive parameters *α_k_* (*k* = 1,2 ⋯ *W*) and *β_t_* (*t* = 1,2 ⋯ *S*) is the critical problem for obtaining $Y_{T}^{j}$ with certain accuracy in order to make $Y_{T}^{j}$ approach $X_{T}^{j}$ as much as possible. Here we take E[(N_j_)^2^] as the difference of $Y_{T}^{j}$ and $X_{T}^{j}$. Then we can obtain Equation (10) as follows [7]:

(10) $$\begin{array}{c} E[{\left(N_{j}\right)}^{2}]=E[{\left((\sum_{k=1}^{W}\alpha_{k}X_{T-k}^{j}+\sum_{p=1}^{S}\beta_{p}X_{T}^{p})-X_{T}^{j}\right)}^{2}]\\ =E[{\left(X_{T}^{j}\right)}^{2}]-2\sum_{k=1}^{W}\alpha_{k}E[X_{T}^{j}X_{T-k}^{j}]-2\sum_{p=1}^{S}\beta_{p}E[X_{T}^{j}X_{T}^{p}]+2\sum_{k=1}^{W}\sum_{p=1}^{S}\alpha_{k}\beta_{p}E[X_{T-k}^{j}X_{T}^{p}]\\ +\sum_{k=1,q=1}^{W}\alpha_{k}\alpha_{q}E[X_{T-k}^{j}X_{T-q}^{j}]+\sum_{p=1,q=1}^{S}\beta_{p}\beta_{q}E[X_{T}^{p}X_{T}^{q}] \end{array}$$

Suppose discrete data $X_{T}^{h}$ and $X_{T}^{t}$ *(h, t* = 1,2 ⋯ *S)* obtained by different nodes at the same time are paired jointly wide sense stationary and set [7]:

(11) $$\Phi_{i}={\left[\alpha_{1}\begin{array}{cc} & \alpha_{2}\cdot \cdot \cdot \alpha_{W}\begin{array}{cc} & \beta_{1}\begin{array}{cc} & \beta_{2}\cdot \cdot \cdot \beta_{S} \end{array} \end{array} \end{array}\right]}^{T}$$

(12) $$c_{x^{j}x^{t}}(k)=E[X_{T}^{j}X_{T+k}^{t}]$$

(13) $$P_{j}=[c_{x^{j}x^{j}}(1)\begin{array}{cc} & c_{x^{j}x^{j}}(2)\cdot \cdot \cdot c_{x^{j}x^{j}}(W) \end{array}\begin{array}{cc} & c_{x^{j}x^{1}}(0) \end{array}\begin{array}{cc} & c_{x^{j}x^{2}}(0) \end{array}\cdot \cdot \cdot c_{x^{j}x^{S}}(0)]$$

(14) $$C_{\mathrm{rr}}^{j}=\begin{array}{cc} C_{x^{j}x^{j}} & C_{x^{j}x^{t}}\\ C_{x^{j}x^{t}}^{T} & C_{x^{t}x^{t}} \end{array}$$

In Equation (14):

(15) $$C_{x^{j}x^{j}}=\begin{array}{cccc} c_{x^{j}x^{j}}(0) & c_{x^{j}x^{j}}(1) & \cdots & c_{x^{j}x^{j}}(W-1)\\ c_{x^{j}x^{j}}(1) & c_{x^{j}x^{j}}(0) & \cdots & c_{x^{j}x^{j}}(W-2)\\ \vdots & \vdots & \cdots & \vdots \\ c_{x^{j}x^{j}}(W-1) & c_{x^{j}x^{j}}(W-1) & \cdots & c_{x^{j}x^{j}}(0) \end{array}$$

(16) $$C_{x^{j}x^{t}}=\begin{array}{cccc} c_{x^{j}x^{1}}(1) & c_{x^{j}x^{2}}(1) & \cdots & c_{x^{j}x^{S}}(1)\\ c_{x^{j}x^{1}}(2) & c_{x^{j}x^{2}}(2) & \cdots & c_{x^{j}x^{S}}(2)\\ \vdots & \vdots & \cdots & \vdots \\ c_{x^{j}x^{1}}(M) & c_{x^{j}x^{2}}(M) & \cdots & c_{x^{j}x^{S}}(M) \end{array}$$

(17) $$C_{x^{t}x^{t}}=\begin{array}{cccc} c_{x^{1}x^{1}}(0) & c_{x^{1}x^{2}}(0) & \cdots & c_{x^{1}x^{S}}(0)\\ c_{x^{2}x^{1}}(0) & c_{x^{2}x^{2}}(0) & \cdots & c_{x^{2}x^{S}}(0)\\ \vdots & \vdots & \cdots & \vdots \\ c_{x^{S}x^{1}}(0) & c_{x^{S}x^{2}}(0) & \cdots & c_{x^{S}x^{S}}(0) \end{array}$$

We can get:

(18) $$E[{\left(N_{j}\right)}^{2}]=c_{x^{j}x^{j}}(0)-2P_{j}^{T}\Phi_{j}+\Phi_{j}^{T}C_{\mathrm{rr}}^{j}\Phi_{j}$$

In order to get predictive coefficients *Φ_j_* when minimizing E[(N_j_)^2^], we can differentiate E[(N_j_)^2^] with respect to *Φ_j_*, and thus get Equation (19):

(19) $$\frac{\partial E[{\left(N_{j}\right)}^{2}]}{\partial \Phi_{j}}=-2P_{j}+2C_{\mathrm{rr}}^{j}\Phi_{j}=0$$

(20) $$\Phi_{j}={\left(C_{\mathrm{rr}}^{j}\right)}^{-1}P_{j}$$

According to the above analysis, if we get certain groups of original data (for example N/3 discussed in DSTD), we can get predictive coefficients *Φ_j_* through Equation (20). Then we can predict the original data obtained by the node at the next moment in the sink. However, data obtained by nodes are time-variant and we must regulate *Φ_j_* in time. Here we let *Φ_j_* move along the opposite direction of the gradient function of E[(N_j_)^2^] to realize the adjustment of *Φ_j_* in time [7], as follows:

(21) $$\Phi_{j}^{\left(n+1\right)}=\Phi_{j}^{n}-\mu \nabla_{j}^{n}$$

We can obtain $\nabla_{j}^{n}$ through Equation (19), *n* stands for certain moment and *μ* stands for the parameter of the opposite direction of the gradient. Because the objective function is convex, we can obtain the minimum value. When we choose *μ* appropriately, we can get the optimal value through Equation (21). The deduction for updating *Φ_j_* goes as follows [7]:

(22) $$\Phi_{j}^{n+1}=\Phi_{j}^{n}-\frac{1}{2}\mu (-2P_{j}+2C_{\mathrm{rr}}^{j}\Phi_{j}^{n})$$

Set:

(23) $$U_{n,j}={\left[X_{n-1}^{j}\begin{array}{cc} & X_{n-2}^{j}\cdots X_{n-M}^{j}\begin{array}{cc} & X_{n}^{1} \end{array} \end{array}\begin{array}{cc} & X_{n}^{2} \end{array}\cdots X_{n}^{S}\right]}^{T}$$

We get:

(24) $$P_{j}=X_{n}^{j}U_{n,j}$$

(25) $$C_{\mathrm{rr}}^{j}=U_{t,j}U_{t,j}^{T}$$

Combining with the following two equations [7]:

(26) $$Y_{n}^{j}={\left(U_{n,j}\right)}^{T}\Phi_{j}^{n}$$

(27) $$N_{n,j}=X_{n}^{j}-Y_{n}^{j}$$

We can get:

(28) $$\Phi_{j}^{n+1}=\Phi_{j}^{n}-\mu U_{n,j}(-X_{n}^{j}+{\left(U_{n,j}\right)}^{T}\Phi_{j}^{n})=\Phi_{j}^{n}+\mu U_{n,j}N_{n,j}$$

So the updating formula of *Φ_j_* can be expressed as follows [7]:

(29) $$Y_{n}^{j}={\left(\Phi_{j}^{n}\right)}^{T}U_{n,j}$$

(30) $$N_{n,j}=X_{n}^{j}-Y_{n}^{j}$$

(31) $$\Phi_{j}^{n+1}=\Phi_{j}^{n}+\mu U_{n,j}N_{n,j}$$

Up to now, we can estimate $Y_{n}^{j}$ by updating coefficient *Φ_j_*. In order to get original data in structure tree, we need the compression code transmitted from the nodes. Now let’s explain how the sink can compute the value of *i*, which is needed by the nodes to compress original data. We can estimate $X_{n}^{j}$ through $Y_{n}^{j}$ under the condition that the distance of neighbor nodes in sub-sequence 2*^i^*^−1^ *Δ* is larger than *N_n,j_*. The mean and variance of *N_n,j_* are 0 and $\delta_{N_{j}}^{2}$ respectively. If 2*^i^*^−1^ *Δ* is larger than *N_n,j_* under the probability *P*, according to the Chebyshev inequality, we can get the value of *i* through the following formula:

(32) $$P\{\left|N_{n,j}\right|<2^{i-1}\Delta \}\geq 1-\frac{\delta_{N_{j}}^{2}}{{\left(2^{i-1}\Delta \right)}^{2}}=P$$

Then we can get:

(33) $$i=\frac{1}{2}\log_{2}^{\frac{\delta_{N_{j}}^{2}}{\left(1-P\right)\Delta^{2}}}+1$$

For a given probability *P*, the sink can compute the value of *i* according to equation (33) and $\delta_{N_{j}}^{2}$ can be initialized by Equation (34) [7]:

(34) $$\delta_{N_{j}}^{2}=\frac{1}{N-1}\sum_{n=1}^{N}N_{n,j}^{2}$$

According to the above analysis, we can describe the DSTD algorithm as follows:

1. The sink obtains N/3 groups of original data transmitted from all nodes and then computes the initial predicted coefficient *Φ_j_* and constructs the structure tree based on the obtained original data (N represents the number of original data groups sensed by node in a complete algorithm cycle)
2. The sink allocates a node in turn to transmit original data and computes the value of *i* for other nodes.
3. The node allocated in (2) transmits original data to the sink. Other nodes get compression order *i* from the sink and compute the compression code through the modulo operator. Then compression codes are transmitted to the sink from the nodes.
4. Combining the predictive coefficient *Φ_j_* in Step (2) with the original data transmitted in Step (3), the sink can get $Y_{n}^{j}$ (corresponding to the estimated value of the original data obtained by node *j* at the moment n). Meanwhile, the compression code obtained from the node can locate a sub-sequence. Together with $Y_{n}^{j}$, the sink can get $X_{n}^{j}$ in the sub-sequence as the optimal estimation of the original data. Then $X_{n}^{j}$ becomes an already known condition for the next node to estimate original data at the same moment. That is to say, the new estimated data should be considered when regulating *Φ_j_*.
5. If original data of all nodes are estimated, the sink will compute and transmit compression order to nodes for the next moment. If the number of groups of original data obtained by nodes is up to N, the algorithm will turn to Step (1) or turn to Step (2).

**Figure 3.**
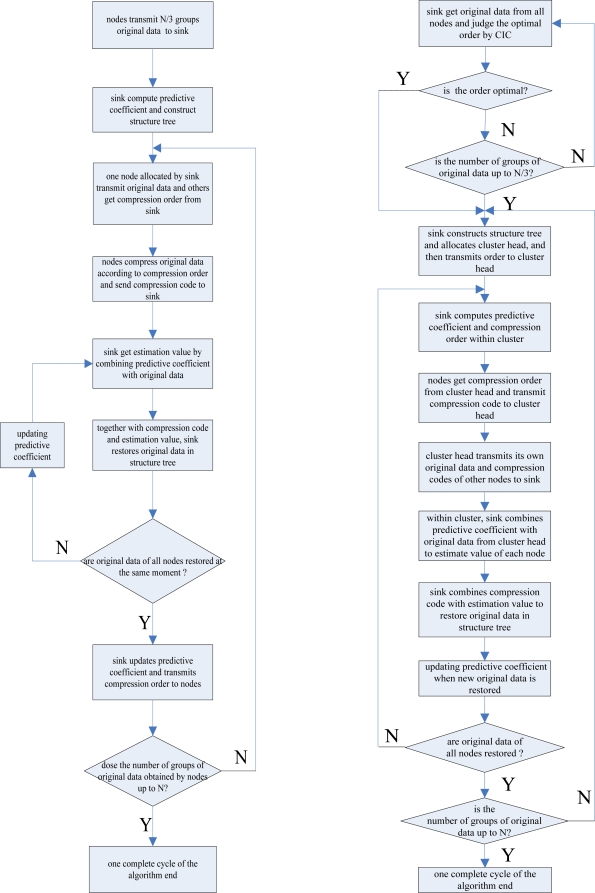
(a) The flow chart of DSTD; (b) The flow chart of COOE-DSTD.

The flow chart of the DSTD algorithm is shown in Figure 3a. According to the theory introduced above, this paper has proposed the COOE-DSTD algorithm, which is illustrated as follows (the flow chart of the COOE-DSTD algorithm is shown in Figure 3b):

1. All nodes transmit original data to the sink and then the sink will judge whether the order is optimal or not by CIC when every cycle transmission is finished. If the order is not optimal and the number of rounds is smaller than N/3, nodes will continue to transmit original data, or the sink will compute the initial predictive coefficient *Φ_m_* and construct the structure tree.
2. The value of the optimal order is related with the predicted estimation coefficients which are associated with the space relativity of the original data obtained from nodes, so the sink divides all nodes into some clusters based on the value of the optimal order. Then the sink allocates a cluster head for every cluster and computes the compression instruction for every node in the cluster.
3. Nodes within a cluster apply the mod operation to the original data based on the compression order for getting the compression code. Then the cluster transmits all compression codes and its own original data to the sink.
4. Within a cluster, the sink combines the predictive coefficient *Φ_m_* in Step (2) with the original data transmitted by the cluster head, and the sink can get $Y_{r}^{m}$ (corresponding to the estimated value of the original data obtained by node m at the moment r). The compression code obtained from the node can locate a sub-sequence. Together with $Y_{r}^{m}$, the sink can ascertain the value $X_{r}^{m}$ in the sub-sequence as the optimal estimation of the original data. Then $X_{r}^{m}$ becomes an available condition for the next node to estimate original data at the same moment. Namely, new estimated data should be considered when regulating *Φ_m_*.
5. If the original data of all nodes are estimated, the ink will compute and transmit the compression order to clusters for the next moment. If the number of groups of original data obtained by the nodes is up to N, the algorithm will turn to Step (1), or, turn to Step (2).

Compared with the DSTD algorithm, optimal order estimation and the operation of clustering are introduced in the COOE-DSTD algorithm, which can decrease the dimension of the data disposed in the sink. The reason is that DSTD algorithm takes all nodes as relative nodes of the node waiting for estimating. If the size of the wireless sensor networks is big enough, some remote nodes which have little relativity with the node waiting for estimating will be considered impertinently, which not only increases the complexity of computation, but also decreases the accuracy of the algorithm.

Contrarily, the COOE-DSTD algorithm only considers those nodes which have spatial-temporal relativity with the node waiting for estimating through the judgment of optimal order estimation, which not only decreases the complexity of computation and the time delay, but also increases the accuracy of the restored data. Meanwhile, the average energy of node can be reduced because the actual data transmitted from nodes to sink is deceased. The method of dividing nodes into clusters is introduced in the COOE-DSTD algorithm, so the sink can locate the place where exceptions have taken place through the cluster structure. Because the predicted coefficients are constructed based on clusters and the data obtained by nodes in the same cluster naturally have spatial-temporal relativity, so the real-time ability and expandability of the whole monitoring system are obviously improved when the COOE-DSTD algorithm is used.

## 4. Simulation Results and Analysis

Presented above is the theoretical analysis of the performance and basic principles of the proposed algorithm. Here we will verify the above analysis through simulation examples. From the flow chart, we know that optimal order estimation and the clustering operation are introduced in the proposed algorithm. Compared with the DSTD algorithm, the main merits of the COOE-DSTD algorithm are shown as follows:

1. As known to all, most node energy is spent on transmitting original data. The number of groups of original data which must be sent to the sink can be reduced through the optimal order estimation, so the average energy cost per node can evidently be reduced.
2. The computing is concentrated on those nodes which have some extent relativity with the node which waits for estimating, and some nodes which have little relativity are ignored, so the data computation dimension is decreased, and then the accuracy of the data can be improved.
3. If the size of wireless sensor networks is very large, after the operation of clustering, sinks can locate the place where exceptions have taken place throughout the cluster structure. Compared with the one by one searching way, the clustering operation can reduce time delays and efficiently improve the real-time ability of the system.

### 4.1. Performance Evaluation Model of the Algorithm

Now the comparison is made between the DSTD algorithm and the COOE-DSTD algorithm through simulation examples. First we should set the criteria for evaluating the performance of the algorithms. Reducing the average energy cost per node to extend the service life of the whole system is one of the most important goals of the proposed algorithm. Besides, the ratio of peak signal to noise is used to evaluate the quality of the restored data in the field of data compression. The COOE-DSTD algorithm is not only able to reduce the communication load to decrease the energy cost of the nodes, but also can guarantee the accuracy of the restored data, so here we take the average energy cost per node, signal to noise ratio and their ratio as performance evaluation criteria for the algorithms. We can refer to Wang’s description in which Strong ARM SA-1100A is taken as an example to calculate the energy cost of nodes. The total energy cost of a node can be expressed as follows [11]:

(35) $$E=E_{\mathrm{lp}}+E_{\mathrm{lt}}+E_{\mathrm{lr}}+E_{\mathrm{rt}}$$

In Equation (35), *E_lp_* stands for the energy cost for computing in a node, and *E_lt_* stands for the energy cost for transmitting data between nodes in the surveillance area, and *E_lr_* stands for the energy cost for receiving data from the sink, and *E_rt_* stands for the energy cost for transmitting data to the sink. The formula for computing the energy cost for transmitting data is shown as follows [11]:

(36) $$E_{\mathrm{lt}}=E_{\mathrm{elec}}*k+{ɛ}_{\mathrm{amp}}*k*D^{2}$$

In the above equation, E_elec_ = 50 nJ/bit, ε_amp_ = 100 pJ/b/m^2^, *K* stands for the number of transmitted bits, D stands for the transmission distance. The calculation of E_rt_ with Equation (36) is similar. The energy cost equation of receiving data is shown as follows [11]:

(37) $$E_{\mathrm{lr}}=E_{\mathrm{elec}}*k$$

The meaning of the parameters in Equation (37) is the same as referred to above [11]:

(38) $$E_{\mathrm{lp}}=\mathrm{IC}*{\mathrm{Vdd}}^{2}$$

In (38), parameter *I* stands for the number of cycles per instruction for the main chip, and *C* stands for the average capacitance switched per cycle, Vdd stands for voltage, and *C* approximates 0.67 nf for the Strong ARM SA-1100A.

For an ordinary processor, if the distance from node to sink is vastly larger than the distance from node to node, the energy cost of executing instructions can be ignored, so the formula of the energy cost can be simplified as follows:

(39) $$E\approx E_{\mathrm{rt}}$$

Both the COOE-DSTD algorithm and the DSTD algorithm need to transmit some groups of original data to the sink in the initialization stage, and then transmit the compression code to the sink, so the energy cost of a node at the beginning of algorithm is larger than later and it is rational to set the average energy cost per node as a performance criterion of the algorithm.

The ratio of peak signal to noise is given by:

(40) $$\mathrm{SNR}=10\log[\frac{\mathrm{AM}}{\sum_{x=0}^{M-1}{\left(f(x)-\hat{f}(x)\right)}^{2}}]$$

In Equation (40), *A* stands for the peak value transmitted by node, *M* stands for the number of group of input data, *f*(*x*) stands for input data, and *f̂*(*x*) stands for output data.

### 4.2. Simulation Experiment

**Figure 4.**
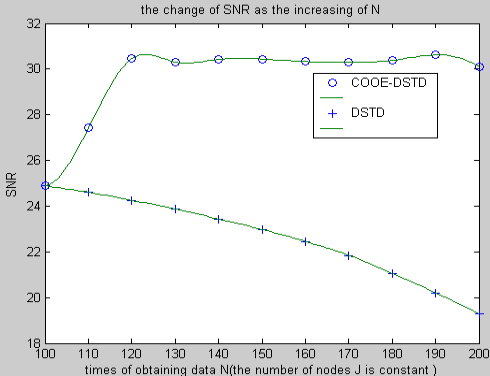
The change of SNR with the increase of N.

Here we suppose nodes are distributed evenly in a surveillance area. The distance from node to neighbor node is 1 meter, and the distance from node to sink is 1,000 meters. Figure 4 shows the SNR performance of the COOE-DSTD algorithm and the DSTD algorithm. From Figure 4, we know that if the number of nodes is constant, the SNR will descend in the DSTD algorithm while it will increase at first and then fluctuate at a value in the COOE-DSTD algorithm as the data obtained by the node in one algorithm cycle increases. According to Figure 4, we know that the accuracy of the restored data is improved in the COOE-DSTD algorithm. The reason for the decrease of the SNR in the DSTD algorithm is that as the obtained data increases in one algorithm cycle, more and more data which have little temporal relativity are imported to estimate the new node value. The problem can be avoided in the COOE-DSTD algorithm through the estimation of optimal order. Data which have little temporal relativity are discarded in the COOE-DSTD algorithm, so the computational complexity is reduced and the accuracy of restored data is improved efficiently.

**Figure 5.**
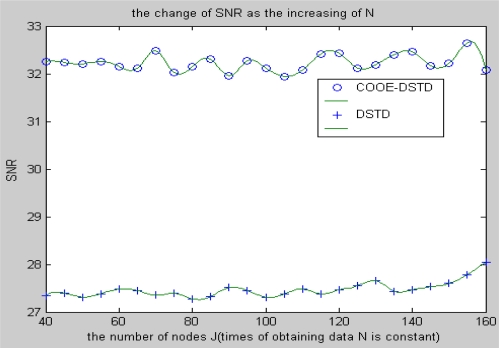
The change of SNR as the increase of nodes J.

From Figure 5, we know that the SNR has slight fluctuations both in the DSTD algorithm and the COOE-DSTD algorithm, but the latter are always larger than the former.

The reason is similar with the factors analyzed above. More and more data which have little spatial relativity are imported to estimate a new node value in the DSTD algorithm as the number of nodes increases. Similarly, through the optimal order estimation, data which have little spatial relativity are discarded in the COOE-DSTD algorithm, so the SNR of the COOE-DSTD algorithm is always larger than that of DSTD algorithm.

**Figure 6.**
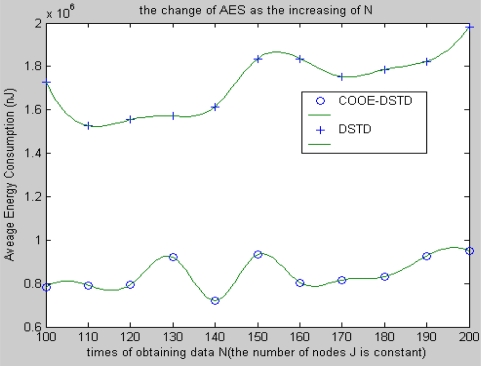
The change of AEC as N increases.

Figure 6 shows that the average energy cost has slight fluctuations both in the DSTD algorithm and the COOE-DSTD algorithm as the data obtained by a node increases in one algorithm cycle, but the latter is always lower than the former. The reason is that through the optimal order estimation, the original data transmitted by a node in the COOE-DSTD algorithm is less than that in the DSTD algorithm.

**Figure 7.**
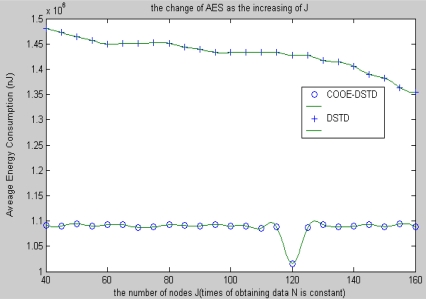
The change of AEC with the increase of nodes J.

From Figure 7, we know that the average energy cost of node in the COOE-DSTD algorithm is less than that in the DSTD algorithm as the nodes increase.

The reason is that all other nodes are taken as relative nodes in the DSTD algorithm when computing the compression order in the sink, so the compression code in the DSTD algorithm is longer than that in the COOE-DSTD algorithm, which leads to the energy cost per node in the DSTD algorithm being larger than that in the COOE-DSTD algorithm. Besides, we can find that the average energy cost per node shows a local minimum when the number of nodes of a sensor network is 120. That is to say, when the size of sensor networks is close to 120 in an application, we can obtain a local optimum by setting the number of nodes at 120 in applying the COOE-DSTD algorithm.

**Figure 8.**
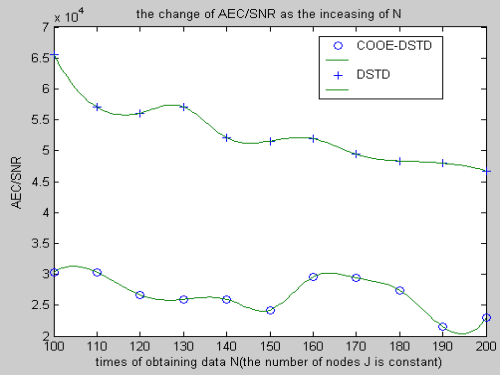
The change of AEC/SNR with the increase of N.

**Figure 9.**
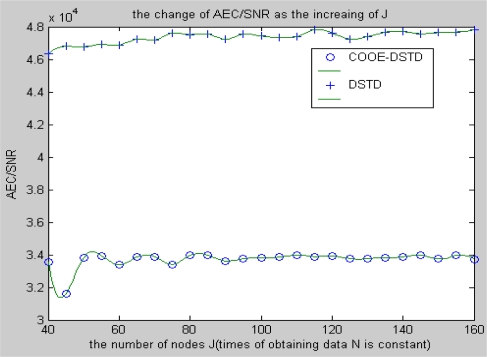
The change of AEC/SNR with the increase of nodes J.

From Figure 8 and Figure 9, we can get similar results with the above simulations when we take AEC/SNR as the performance criteria (AEC means average energy cost). Slight fluctuations can be found in both the DSTD algorithm and the COOE-DSTD algorithm and the former is always larger than the latter as the data obtained by nodes in one algorithm cycle increases.

The simulation result of the situation of increasing the number of nodes is similar with the situation in Figure 8. The actual computational cost is reduced by introducing optimal order estimation and the operation of clustering, so the average energy cost is decreased and the accuracy of restored data is evidently improved.

## 5. Conclusions

With the features of wireless sensor networks in mind, we have proposed the COOE-DSTD algorithm through implementation of optimal order estimation and the operation of clustering. Compared with the DSTD algorithm, the actual amount of computing is reduced because the optimal order is obtained, so the accuracy of the restored data can be greatly improved and the average energy cost can be reduced efficiently. The operation of clustering enables the sink to locate nodes which have detected exceptions quickly, therefore the time delay of the system can be greatly reduced and the scalability of the system will evidently be improved. However, the algorithm proposed in this paper mainly aims to deal with one-dimensional data. In future work we will try to extend the algorithm to deal with two-dimensional data.
